# Social determinants of health and diabetic foot disease

**DOI:** 10.1186/1757-1146-8-S2-O36

**Published:** 2015-09-22

**Authors:** Adrian Singh, Peter Lazzarini, Lloyd Reed, Gavin Turrell

**Affiliations:** 1 School of Public Health and Social Work, Queensland University of Technology, Brisbane, Queensland, 4059, Australia; 2 Institute of Urban Indigenous Health, Brisbane, Queensland, 4006, Australia; 3 School of Clinical Sciences, Queensland University of Technology, Brisbane, Queensland, 4059, Australia; 4 Institute of Health and Biomedical Innovation, Queensland University of Technology, Brisbane, Queensland, Australia; 5 Allied Health Research Collaborative, Metro North Hospital & Health Service, Brisbane, Queensland, Australia

**Keywords:** Social Determinants of Health, Diabetic Foot Disease, Lower Extremity Amputation

## Background

Diabetic foot disease (DFD) is the leading cause of hospitalisation and lower extremity amputation (LEA) in people with diabetes. Many studies have established the relationship between DFD and clinical risk factors, such as peripheral neuropathy and peripheral arterial disease. Other studies have identified the relationship between diabetes and non-clinical risk factors termed social determinants of health (SDoH), such as socioeconomic status. However, it appears very few studies have investigated the relationship between DFD and SDoH. This paper aims to review the existing literature investigating the relationship between DFD and the SDoH factors socioeconomic status (SES), race and geographical remoteness (remoteness).

## Process

Electronic databases (MEDLINE, CINAHL, and PubMed) were searched for studies reporting SES, race (including Aboriginal and Torres Strait Islander in Australia) and remoteness and their relationship to DFD and LEA. Exclusion criteria were studies conducted in developing countries and studies published prior to 2000.

## Findings

Forty-eight studies met the inclusion criteria and were reviewed; 10 in Australia. Overall, 28 (58%) studies investigated LEA, 10 (21%) DFD, and 10 (21%) DFD and LEA as the DFD-related outcome. Thirty-six (75%) studies investigated the SDoH risk factor of race, 22 (46%) SES, and 20 (42%) remoteness. SES, race and remoteness were found to be individually associated with LEA and DFD in the majority of studies. Only four studies investigated interactions between SES, race and remoteness and DFD with contrasting findings. All four studies used only LEA as their investigated outcome. No Australian studies investigate the interaction of all three SDoH risk factors on DFD outcomes.

## Conclusions

The SDoH risk factors of SES, race and GR appear to be individually associated with DFD. However, only few studies investigated the interaction of these three major SDoH risk factors and DFD outcomes with contrasting results. There is a clear gap in this area of DFD research and particularly in Australia. Until urgent future research is performed, current practice and policy does not adequately take into consideration the implication of SDoH on DFD

